# Rhabdomyolysis following cardiac surgery: from prevalence to prevention

**DOI:** 10.1186/cc13384

**Published:** 2014-03-17

**Authors:** AS Omar, HA Ewila, SR Aboulnaga, AK Tuli

**Affiliations:** 1 Hamad Medical Corporation, Doha, Qatar; 2 Beni Suef University, Egypt; 3 Suez Canal University, Egypt; 4 Ain Shams University, Egypt

## Introduction

In the view of robust consequences following cardiac surgery, acute kidney injury (AKI) remains a major concern. Rhabdomyolysis (RML) following cardiac surgery and its relation to AKI need to be investigated. We aim to study the prevalence of RML development following cardiac surgery and the perioperative risk factors that may expedite the occurrence of RML.

## Methods

All patients undergoing cardiac surgeries in our hospital were enrolled in the study during the period of 1 year in a prospective descriptive study measuring the occurrence of RML and its association with AKI, where all patients in the study underwent serial assessment of serum creatinine kinase (CK) and serum myoglobin. Serial renal function, prior statin treatment, cardiac injury, lengths of ventilation, and lengths of stay in the ICU and hospital were monitored.

## Results

We recruited 202 patients in our study, 185 males and 17 females with mean age 52 ± 12.4 years. According to the existence of RML (CK 2,500 U/ml or more) [1], patients were divided into group 1 where RML was identified in 17 patients (8.4%), which was associated with AKI in seven patients (41%), and group 2 without RML (185 patients), where AKI occurred in 34 patients (18.4%) (*P *= 0.025). We observed a significantly longer duration of ventilation and lengths of stay in the ICU and in hospital in the RML group (*P *< 0.01 for all observations).

## Conclusion

Early increase in the serum CK and myoglobin in postoperative high-risk cardiac surgeries may predict the concomitance of early AKI, where proper intervention may prevent the sequelae of logistic organ dysfunction.
